# Vision and sensorimotor defects associated with loss of Vps11 function in a zebrafish model of genetic leukoencephalopathy

**DOI:** 10.1038/s41598-022-07448-1

**Published:** 2022-03-03

**Authors:** Shreya Banerjee, Lillian E. Ranspach, Xixia Luo, Lauren T. Cianciolo, Joseph Fogerty, Brian D. Perkins, Ryan Thummel

**Affiliations:** 1 Department of Ophthalmology, Visual and Anatomical Sciences, Wayne State University School of Medicine, Detroit, MI USA; 2 Department of Ophthalmic Research, Cole Eye Institute, Cleveland Clinic, Cleveland, OH USA

**Keywords:** Behavioural genetics, Development, Neurodevelopmental disorders, Oligodendrocyte, Neurodegeneration, Development of the nervous system, Myelin biology and repair, Oculomotor system, Sensorimotor processing, Reflexes

## Abstract

Genetic Leukoencephalopathies (gLEs) are heritable white matter disorders that cause progressive neurological abnormalities. A founder mutation in the human endolysosomal trafficking protein *VPS11* has been identified in Ashkenazi Jewish patients manifesting classic gLE symptoms of hypomyelination, developmental delay, motor and systemic deficits. In this study, we characterized the visual and sensorimotor function of two zebrafish *vps11* mutant lines: the previously reported *vps11(plt)*, and a new *vps11*(−/−) null mutant line, using behavioral analysis to track larval motor responses to visual and acoustic stimuli. We found that mutant larvae from both *vps11*(*plt*) and *vps11*(−/−) lines were able to visually distinguish light and dark, but showed a progressive loss of a normal sensorimotor response to visual stimuli from 5 days post fertilization (dpf) to 7dpf. Additionally, optokinetic response analysis performed at 5dpf indicated that the mutants were significantly visually impaired. Both mutant lines also displayed a progressively lower sensorimotor response to a singular acoustic stimulus from 5-7dpf. Next, we tested the habituation response of the mutant lines to series of acoustic taps. We found both mutant lines habituated faster than their siblings, and that *vps11(plt)* mutants habituated faster than the *vps11*(−/−) mutants. Together, these data suggest that loss of Vps11 function results in progressive visual and sensorimotor abnormalities in the zebrafish *vps11(plt)* and *vps11*(−/−) mutant lines. This is the first study to characterize behavioral deficits in a vertebrate model of Vps11-dependent gLE. The mutants and behavioral assays described here could be a valuable model system in which to test potential pharmacological interventions for gLE.

## Introduction

Genetic leukoencephalopathies (gLEs) are heritable disorders of the white matter with primary neuronal involvement^1^. Patients with gLE manifest heterogeneous neurological and systemic symptoms, which may include motor impairment, hypotonia, ataxia, cortical visual impairment, ophthalmologic abnormalities, cognitive deficit and seizures^2^. Magnetic resonance imaging (MRI) is the major diagnostic approach to these white matter abnormalities. However, given the progressive and variable nature of the disease phenotypes, a definitive diagnosis is often a challenge^3^. Recent advances in whole exome sequencing (WES) now permit more definite diagnoses for gLE and for the discovery of novel genetic mutations that result in gLE^3,4^. WES was recently used to identify a novel mutation in *Vacuolar Protein Sorting 11* (*VPS11*) in patients with gLE^5,6^. The autosomal recessive mutation *VPS11:C846G* results in infantile onset of gLE characterized by hypomyelination and developmental delay, along with classic gLE hallmarks of progressive motor and sensory system deficiencies^6^. With a high carrier frequency of 1:250 in Ashkenazi Jewish (AJ) families, this missense mutation was hypothesized to arise from a founder effect^6^. Biochemical in vitro analysis revealed that the truncated mutant protein has lower expression, faster turnover, and impairments in autophagy flux^6^; however, the exact mechanism that leads to hypomyelination and disease pathology is unclear.

**Figure 1:**
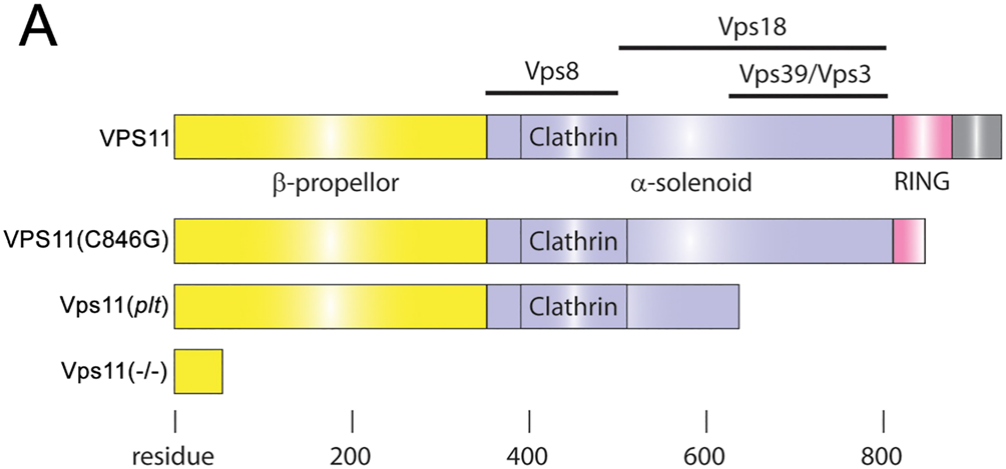
Schematic representation comparing the lengths and interaction domains of human VPS11 and VPS11(C846G) with zebrafish Vps11(plt) and Vps11(−/−). (**A**) Top: Schematic representation of full-length human VPS11 protein, showing multiple highly-conserved protein domains. Lines above show the putative interactive domains with other Vps effectors based largely on work done in yeast. Below: Schematic representations of human VPS11(C846G), zebrafish Vps11(plt), and zebrafish Vps11(−/−), resulting in presumptively shorter truncated protein products. Note that zebrafish and human sequences do not show 100% nucleic acid identity, but do show conserved protein domains.

Vps11, Vps16, Vps18 and Vps33 form a C-Vps protein complex that is evolutionally conserved back to yeast^7,8^. The C-Vps protein complex is the backbone of two tethering complexes: Homotypic Fusion and Protein Sorting (HOPS) and Class C-core Vacuole/Endosome Tethering (CORVET). These highly conserved complexes facilitate the tethering and fusion of vesicles within the endolysosomal and autophagy pathways^8,9^. The Vps11 protein contains a β-propeller at the N-terminus, a RING domain at the C-terminus, and an α-solenoid in between (Fig. 1). Based on extensive studies in yeast, putative interactions between VPS11 and VPS18, VPS39, VPS3, and VPS8 are thought to be conserved in vertebrates (Fig. 1). Zhang et al., 2016^6^ reported a missense mutation in human *VPS11* causing a cysteine at the 846 residue in the RING domain to be converted to glycine. In vitro analysis suggested that this mutation resulted in a truncated protein with reduced stability and ability to bind VPS18.

The first example of a vertebrate with impaired Vps11 function was the *vps11 platinum (plt)* mutation identified in zebrafish^10^. An additional zebrafish mutant has also been identified^11,12^. The *vps11(plt)* allele contains a single base change and single base insertion resulting in a premature stop codon (Fig. 1). The mutation presumably produces a truncated Vps11(plt) protein, but this has not been directly tested. The *vps11(plt)* zebrafish mutants exhibited hypopigmentation in the body and retinal pigmented epithelium (RPE), hepatomegaly, RPE and photoreceptor degeneration, and juvenile death^10^. A follow-up report showed that the loss of Vps11 function in the *plt* phenotype also resulted in increased neuronal cell death and myelination defects^6^, which suggested that it could be utilized as a model for Vps11-dependent gLE. However, it is presently unclear whether the *vps11*(*plt*) zebrafish mutant line elicits progressive motor and visual system pathologies as observed in human patients with the *VPS11:C846G* mutation.

In this study, we characterized the visual and sensorimotor function of two zebrafish *vps11* mutant lines: the previously reported *vps11(plt)*, and a new *vps11*(−/−) null mutant line we generated for these studies. Specifically, we subjected larval zebrafish from these lines to behavioral studies to elucidate their vision and sensorimotor function. We found that both *vps11*(*plt*) and *vps11*(−/−) mutant larvae could visually distinguish light and dark, but showed a progressive loss of sensorimotor responses to visual stimuli from 5 days post fertilization (dpf) to 7dpf. In addition, optokinetic response analysis at 5dpf indicated that larvae from both mutant lines were significantly visually impaired. Next, we tested motor function apart from the visual system in the mutant larvae using an acoustic/tap startle response. Again, we found that larvae from both mutant lines displayed a progressively reduced sensorimotor response to a single acoustic stimulus from 5-7dpf. Finally, as an initial assessment of higher circuit development, we tested the habituation response in the mutant lines in response to series of acoustic taps. Interestingly, both mutant lines habituate faster than their siblings, and the *vps11(plt)* mutants habituate faster than *vps11*(−/−) mutants. Together, these data demonstrate progressive visual system and sensorimotor abnormalities in the zebrafish *vps11(plt)* and *vps11*(−/−) mutant lines. This study represents the first report of behavioral deficits in a vertebrate model of Vps11-dependent leukoencephalopathy. With the discovery Vps11 as the novel causative gene of gLE, these zebrafish models allow for further exploration of how abnormal Vps11 leads to progressive loss of visual and motor function.

## Methods

### General statement

All methods were carried out in accordance with relevant guidelines and regulations, and all animal studies were in accordance with ARRIVE guidelines (https://arriveguidelines.org).

### Zebrafish lines and maintenance

Three zebrafish lines were used in this study: AB (wild-type), *vps11(plt)*^*wsu1*^ ^10^ and *vps11*(−/−)^*wsu3*^ mutant line. Note that both the *vps11(plt)* and *vps11*(−/−) mutant lines were created and maintained on the AB background. Fish were fed a combination of brine shrimp and dried flake food three times daily and maintained at 28.5 °C on a 14 h light (250 lx): 10 h dark cycle^13^. All animal care and experimental protocols used in this study were approved by the Institutional Animal Care and Use Committee at Wayne State University School of Medicine and at the Cleveland Clinic.

### Generation of the ***vps11***(−/−) mutant line

The *vps11*(−/−) mutant line was generated using transcription activator-like effector nucleases (TALENs). A pair of forward and reverse XTN TAL Nuclease Plasmids were designed and synthesized by Transposagen Biopharmaceuticals, Inc (Lexington, KY) to target a *Bam*HI restriction site within the first 50 nucleotides of the coding sequence of exon 1 of *vps11*. The target sequence (underlined) was as follows: 5’ – TTTTCGATAAAGAAACTGTGAAGGATCCGAGCGAAAATGGGAAAAACTTCCA – 3’. Primers designed around the *Bam*HI restriction site can be used for genotyping, as the restriction site was disrupted in the mutant animals. The TALEN plasmids were linearized, mRNA synthesis was performed with mMACHINE T7 Ultra Kit followed by polyA addition (ab1345, Ambion), and synthesized mRNA was purified using Spin Column Chromatograph (Catalogue# 11,814,427,001, Roche). Equal volumes of forward and reverse mRNAs were mixed to a final concentration of 50 ng/uL each and ~ 5nL of the TALEN mRNA mixture (100 pg/nL) was micro-injected into 1-cell-stage wild-type embryos (AB strain). Non-homologous end joining repair events were confirmed the following day in a subset of injected embryos by PCR/restriction analysis. Cohorts of successful injections were raised for 1 month, at which point F0 mutant carriers were identified from genotype sequencing of individual tail fin snips. Heterozygous mutants were raised to sexual maturity and bred to wild-type fish to verify germline transmission. Mating between heterozygous pairs carrying the exact same mutation were performed to obtain the *vps11* null mutant line. Phenotypic analysis determined that the new homozygous *vps11*(−/−) mutants exhibit the classic characteristics of the *vps11(plt)* mutants, namely: hypopigmentation, hypomyelination, and CNS apoptosis (Suppl. Fig. 1).

### Behavioral analysis

Fish were bred from heterozygous parents and larvae were raised under standard conditions. Homozygous mutant larvae were readily distinguished from their siblings at 3dpf due to their hypopigmentation phenotype^10^. At 4 or 6dpf, individual larvae were placed in wells of a 24-well plate (diameter 1.65 cm wells) to be acclimatized for 24 h before behavior analysis (12 mutant larvae and 12 sibling control larvae per 24-well plate). Behavior analysis was performed on the following day between 1 and 4 PM using the DanioVision Observation Chamber (Noldus Information Technology, Wageningen, Netherlands) linked with the EthoVision XT13 software. The software package allowed us to create stimulus paradigms that automatically controlled the lighting conditions, acoustic stimuli, and recording times. In addition, the DanioVision Observation Chamber was linked to the DanioVision Temperature Control Unit, which supplied a steady flow of water to the chamber and maintained the larvae at constant temperature of 28.0 ± 0.5 °C during the procedure. Finally, the larvae were live-tracked using the EthoVision XT13 software via a Basler Gen1 Camera (Basler acA1300-60). Camera resolution was set at 1280 × 960 and the frame rate was set at 25. Two stimulus paradigms were used for this study, described in detail below.

*Light/dark paradigm*: Once placed inside the DanioVision Observation Chamber, larvae were subjected to a 12-min acclimation period in the dark, followed by four alternating periods of 3-min light (10,500 lx) and 3-min dark. A light environment was provided by uniform illumination of the entire base panel of the chamber on which the 24-well plate rested. Response to stimulation was recorded as distance moved (cm) and velocity (cm/s) during each 3-min stimulus period. Heat maps were generated using the EthoVision XT13 software for every 3-min stimulus period that tracked the total movement of individual larva during that particular stimulus interval. The total distance moved (cm) and average velocity moved (cm/s) for each individual larva was calculated during each 3-min period of light or dark. Statistical differences between the mutants and siblings during the first round of light and dark conditions was calculated using a Student’s t-test with a p < 0.05 as a cutoff for significance.

*Acoustic/tap paradigm*: The acoustic/tap paradigm utilized “taps” administered directly to the base of the DanioVision Observation Chamber. The DanioVision system is pre-installed with taps at 8 intensity levels. The magnitude of the taps is internally consistent within a manufacturer unit, but can vary between units due to slight variations in mounting the Tapping Device. Therefore, we first determined the minimum intensity in our unit that would produce a consistent startle response in wild-type (AB strain) larva, which was intensity 3 (data not shown). This setting was used as the “Low Intensity Tap”. The maximum intensity level of 8 was selected as the “High Intensity Tap”. Once placed inside the DanioVision Observation Chamber, mutant and sibling larvae were subjected to a 12-min acclimation period with no stimulation, followed by either a single tap or multiple taps. For single taps, movement was recorded for 1-min of no stimulation, a single tap at low or high intensity, and then an additional 1-min of no stimulation. For multiple taps, movement was recorded for 1-min of no stimulation, 10 consecutive high intensity taps at 10-s intervals, and then an additional 1-min of no stimulation. Response to stimulation was recorded as distance moved (cm) at each tap event for individual larva. For single taps statistical differences between the mutants and siblings was calculated using a Student’s t-test with a p < 0.05 as a cutoff for significance. For multiple taps, the habituation curves were calculated using GraphPad and statistical differences within and between the groups was determined via a one phase exponential decay as previously described^14^. In addition, the median distance travelled during the series of taps was calculated using GraphPad and statistical differences between the two groups was analyzed using a Welch’s t-test as previously described^14^.

### Optokinetic response

All tests were conducted on 5dpf mutant and sibling larvae between 12 and 6 pm using a VisioTracker 302,060 (TSE Systems, Bad Homburg, Germany) as previously described^15^. Briefly, larval fish were immobilized in 3% methylcellulose preheated to 28.5 °C in 35 mm Petri dishes. All stimuli were presented for 3-s before reversing direction for 3-s. For contrast tests, the stimulus contrast was varied in stepwise increments while maintaining the spatial frequency at 0.06 cycles/degree and the velocity at 7.5 degree/sec. For velocity tests, the stimulus velocity varied between 1.5 and 7.5 degree/sec while maintaining the frequency at 0.06 cycles/degree and the contrast at 70%. The OKR gain, being the ratio of eye:stimulus angular velocity, was plotted as a function of either contrast or velocity. OKR results from sibling larva (n = 14), *vps11(plt)* (n = 6), and *vps11−/−* (n = 4) were analyzed with GraphPad Prism 9.0 using 2-way ANOVA with Sidak’s multiple comparisons test.

## Results

### Tracking the behavioral response to alternating light/dark periods shows that wild-type (AB) 7dpf larvae move significantly more in the in dark periods

Zebrafish larval locomotion has been reported to be influenced by visual based photic stimulation^16^. To establish the visual stimulation paradigm for this study, behavioral analysis of wild-type larva (AB strain) was performed using the Noldus DanioVision system (Suppl. Fig. 2A–B). This system is equipped with both dark/light and acoustic/tap stimuli, a Temperature Control Unit to adjust water temperature, a Basler Gen1 Camera, and EthoVision XT13 software to track larval movements (Suppl. Fig. 2A). We first tested individual wild-type AB larvae at 7dpf, which were placed inside a 24-well plate (Suppl. Fig. 2B) and subjected to a visual stimulation of 4 alternating cycles of light and dark, each lasting 3 min. Larval behavior in response to light/dark cycles was recorded and heat maps generated by the EthoVision XT13 software. Consistent with previous reports^16^, we observed increased movement of the larvae in dark versus light conditions (Suppl. Fig. 2C). During each dark period, the distance travelled and average velocity of movement were significantly higher when compared with each light period (Suppl. Fig. 2D-F). Importantly, these findings were highly reproducible, laying the framework for a robust, unbiased, and sensitive testing paradigm for the *vps11* mutant lines.

### *vps11(plt)* mutants show a progressive loss of normal sensorimotor response to visual stimuli

**Figure 2:**
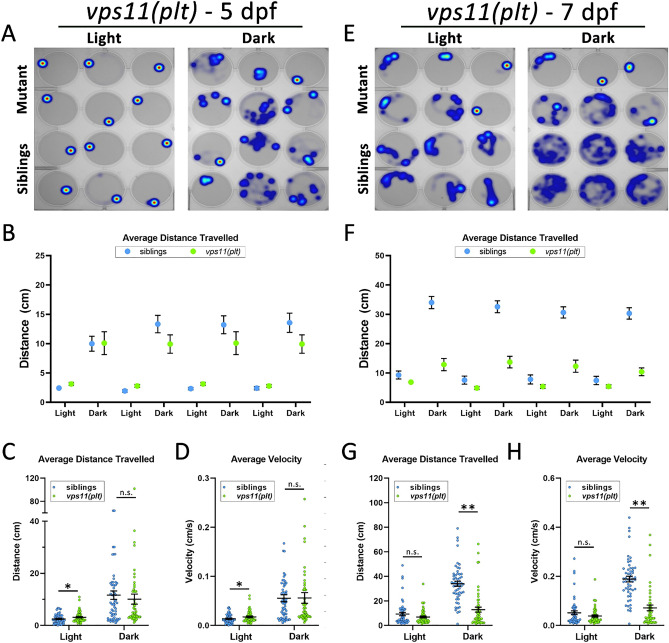
*vps11(plt)* mutants show normal distance travelled and velocity at 5dpf and significantly reduced distance travelled and velocity at 7dpf during alternating cycles of light and dark. (**A**) Representative heat maps of total distance moved by individual larvae at 5dpf (*vps11(plt)* mutants = top two rows; siblings = bottom two rows) during a 3-min period of light and dark. (**B**) Graph representing average distance travelled in 4 alternating light–dark cycles of 3 min each (sibling larvae in blue; *vps11(plt)* mutants in green). (**C**) Graph representing average distance travelled in light and dark periods. (**D**) Graph representing average velocity of sibling larvae and *vps11(plt)* in light and dark periods. n = 60 sibling and 60 *vps11(plt)* larvae at 5 dpf. (**E**) Representative heat maps displaying total distance moved by individual larvae at 7dpf (*vps11(plt)* mutants = top two rows; siblings = bottom two rows) during a 3-min period of light and dark. (**F**) Graph representing average distance travelled by larvae in 4 alternating light–dark cycles of 3 min each (sibling larvae in blue; *vps11(plt)* mutants in green). (**G**) Graph representing average distance travelled in light and dark periods. (**H**) Graph representing average velocity of sibling larvae and *vps11(plt)* in light and dark periods. n = 54 sibling and 52 *vps11(plt)* larvae. *P < 0.05, **P < 0.01. Error bars indicate SEM.

As an initial test of the visuomotor development in *vps11(plt)* mutants, mutant larvae and their siblings were subjected to the aforementioned alternating light/dark periods at 5dpf and 7dpf. At 5dpf, all larvae exhibited greater movement in the dark periods compared with the light periods (Fig. 2A–B). Comparing the movement of the larvae showed that *vps11(plt)* mutants travelled a significantly greater distance and with greater velocity than their siblings during the light periods (Fig. 2C–D). No significant differences in distance moved or velocity were observed during the dark periods (Fig. 2C–D). From 5-7dpf, sibling larvae showed a dramatic increase in distance travelled and velocity in both the light and dark periods.

However, *vps11(plt)* mutants failed to increase their movement to the extent of their siblings during this same time window. As a result, heat maps showed a discernable overall lower motility of the mutant larvae at 7dpf (Fig. 2E–F). In addition, quantification of these movements showed that 7dpf *vps11(plt)* mutants travelled a significantly shorter distance (Fig. 2G) and decreased velocity (Fig. 2H) compared with siblings in each dark period. These data demonstrate that *vps11(plt)* mutants responded to alternating light/dark periods and, at 5dpf, traveled greater or equal distances to their siblings during these periods. However, at 7dpf, *vps11(plt)* mutants moved significantly less than their siblings, suggesting that the sensorimotor response to these visual stimuli becomes progressively more defective during this window of development.

### ***vps11***(−/−) mutants show a progressive loss of normal sensorimotor response to visual stimuli

**Figure 3:**
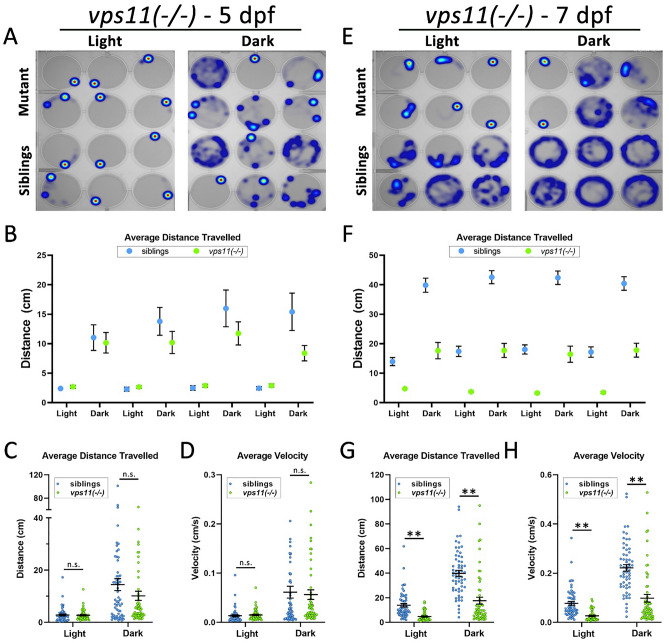
*vps11*(−/−) mutants show normal distance travelled and velocity at 5dpf and significantly reduced distance travelled and velocity at 7dpf during alternating cycles of light and dark. (**A**) Representative heat maps of total distance moved by individual *vps11*(−/−) larvae at 5dpf (mutants = top two rows; siblings = bottom two rows) during a 3-min period of light and dark. (**B**) Graph representing average distance travelled in 4 alternating light–dark cycles of 3 min each (sibling larvae in blue; mutants in green). (**C**) Graph representing average distance travelled in light and dark periods. (**D**) Graph representing average velocity of sibling and *vps11*(−/−) larvae in light and dark periods. n = 60 siblings and 58 *vps11*(−/−) larvae. (**E**) Representative heat maps generated of total distance moved by individual *vps11*(−/−) larvae at 7dpf (mutants = top two rows; siblings = bottom two rows) in a 3-min period of light and dark. (**F**) Graph representing average distance travelled in 4 alternating light–dark cycles of 3 min each (sibling larvae in blue; *vps11*(−/−) mutants in green). (**G**) Graph representing average distance travelled in light and dark periods. (**H**) Graph representing average velocity of sibling and *vps11*(−/−) larvae in light and dark periods. n = 60 siblings and 57 *vps11*(−/−) larvae. **P < 0.01. Error bars indicate SEM.

We next tested our newly-generated *vps11*(−/−) null mutant zebrafish to the same alternating light–dark periods at 5dpf and 7dpf. Similar to the *vps11(plt)* mutants, at 5dpf both the *vps11*(−/−) mutants and their siblings responded to light/dark changes and moved more during the dark periods (Fig. 3A–B). There were no significant differences between *vps11*(−/−) mutant and sibling larvae in regard to average distance travelled (Fig. 3C) and velocity (Fig. 3D) in either light or dark periods at 5dpf. At 7dpf, both sibling and mutant fish showed an increase in distance travelled and velocity. However, *vps11*(−/−) mutants did not increase to the extent of their siblings. Heat maps at 7dpf indicated a discernable lower motility for *vps11*(−/−) mutants compared with their siblings in both light and dark periods (Fig. 3E–F). Quantitative analysis confirmed that the *vps11*(−/−) traveled a significantly shorter distance (Fig. 3G) and lower velocity (Fig. 3H) in both light and dark periods compared with their siblings. These data suggest that *vps11*(−/−) mutant zebrafish show a similar progressive visuomotor defect as the *vps11(plt)* mutants. Notably, in both mutant lines, movement and velocity increased from 5 to 7dpf, but not to the degree of sibling animals during this same time interval.

### ***vps11(plt)*** and ***vps11***(−/−) mutants are significantly visually impaired

**Figure 4:**
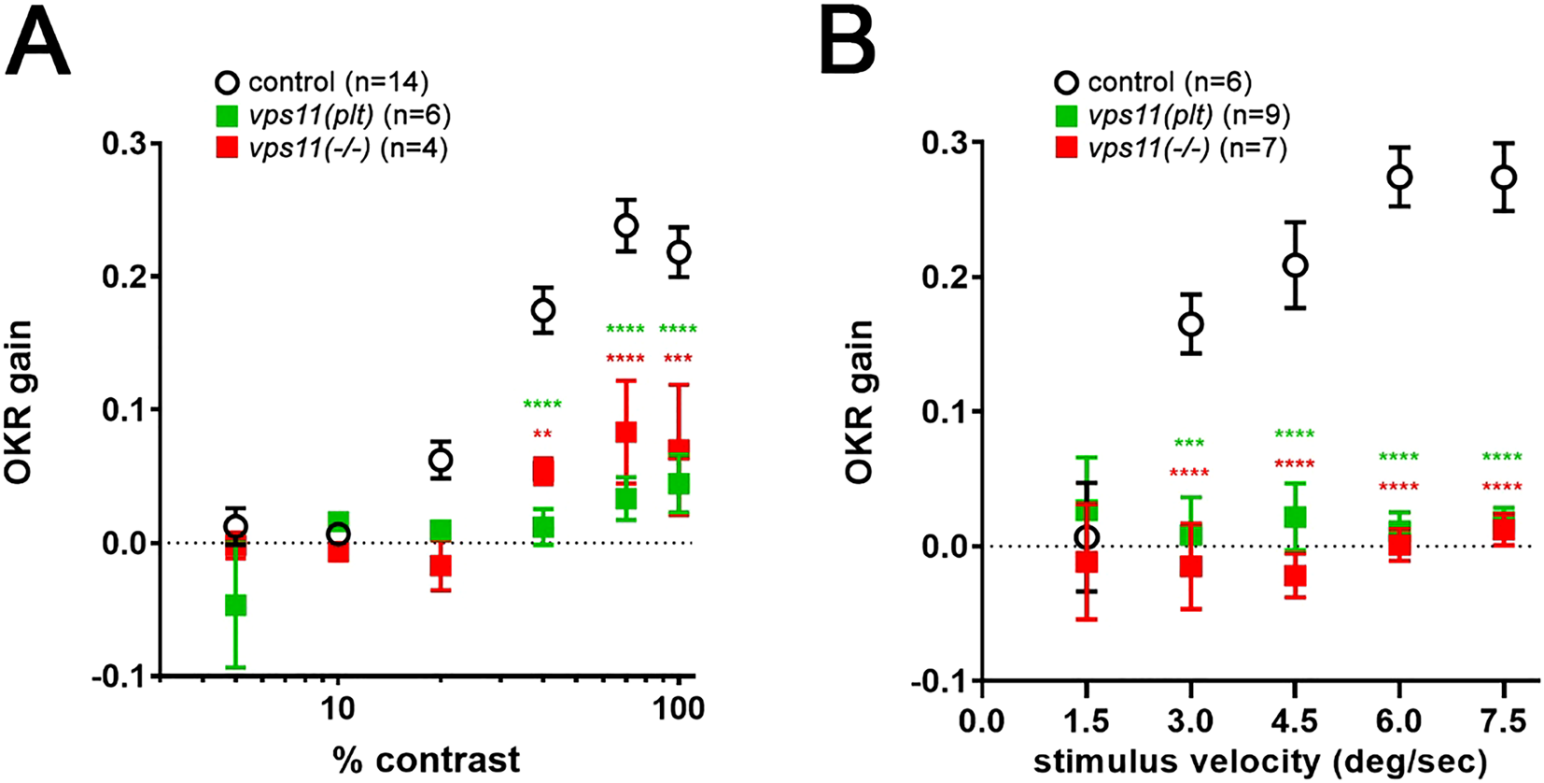
Visual function is compromised in *vps11(plt)* and *vps11*(−/−) mutants. Optokinetic response of larval *vps11(plt)* and *vps11*(−/−) homozygotes compared with sibling controls as a function of (**A**) contrast and (**B**) stimulus velocity. Asterisks indicate significant differences from controls (2-way ANOVA with Sidak’s multiple comparison test) **P < 0.01, ***P < 0.001, ****P < 0.0001.

Both *vps11(plt)* and *vps11*(−/−) mutant larvae responded to light and dark environments, but it is unclear whether they have normal visual acuity. In order to test this, we used the VisioTracker to measure the optokinetic (OKR) response of *vps11(plt)*, *vps11*(−/−) and their siblings at 5dpf. Specifically, *vps11(plt)* and *vps11*(−/−) larvae were stimulated with a black and white grating, moving with a constant velocity of 7.5 degree/s and having a constant spatial frequency of 2.06 cycles/degree. The OKR gain was measured for the larvae while changing contrast levels of the black/white boundary, ranging from 5 to 100%. Even at 100% contrast, both *vps11(plt)* and *vps11*(−/−) larvae showed severely impaired acuity when compared with sibling controls (Fig. 4A).

Next, we modified the OKR setup to test visual response at different stimulus velocities, since it was possible that the constant velocity of 7.5 degree/s was too fast for the hypomyelinated mutants to follow. We repeated the OKR at a constant contrast (70%) and spatial frequency (2.06 cycles/degree), and increasing stimulus velocities ranging from 1.5 to 7.5 degrees/sec. At lower velocity levels, both *vps11(plt)* and *vps11*(−/−) larvae displayed an OKR gain near zero, while sibling control fish exhibited strong OKR gain at 3.0 dg/sec (Fig. 4B). Together, these data indicate that both *vps11(plt)* and *vps11*(−/−) larvae are significantly visually impaired by 5dpf.

### ***vps11(plt)*** and ***vps11***(−/−) mutants show a progressively lower response to an acoustic/tap startle response from 5 to 7dpf

**Figure 5:**
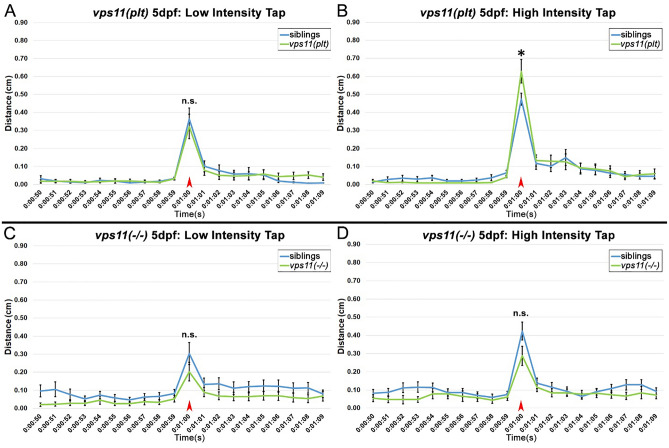
*vps11(plt)* and *vps11*(−/−) mutants at 5dpf show normal distance travelled in response to both low and high intensity acoustic/tap stimuli. (**A**–**D**) Line graphs representing distance travelled by larvae at 5dpf before and after an acoustic/tap stimulus (red arrow). (**A**) Response of *vps11(plt)* (green; n = 55) and sibling larvae (blue; n = 61) to a low intensity stimulus. (**B**) Response of *vps11(plt)* (green; n = 63) and sibling larvae (blue; n = 67) to a high intensity stimulus. (**C**) Response of *vps11*(−/−) (green; n = 59) and sibling larvae (blue; n = 60) to a low intensity stimulus. (**D**) Response of *vps11*(−/−) (green; n = 57) and sibling larvae (blue; n = 60) to a high intensity stimulus. Error bars indicate SEM.

Visual stimulation resulted in a lower motor response in *vps11(plt)* and *vps11*(−/−) larvae at 7dpf (Figs. 2, 3), however, it was unclear whether this progressive defect was due to aberrations in the visual system or motor system (or both). Thus, we used a non-visual cue of a single acoustic/tap stimulus to study the resultant motor response. Using pre-set intensities in the system, we first determined that intensity “3” was the minimum intensity that would elicit a consistent startle response in AB wild-type larvae (*data not shown*). Next, a single-tap stimulus at pre-set intensity 3 (i.e. low intensity) was administered on *vps11(plt)* and *vps11*(−/−) and their respective siblings at 5dpf (Fig. 5A,C). Both mutant lines and their siblings responded to the single-tap acoustic stimulus with a burst of movement immediately following the tap (Fig. 5A,C). Notably, there was no significant difference in the distance moved between the mutant lines and their siblings (Fig. 5A,C). Next, we repeated the test with a new cohort of 5dpf larvae using pre-set intensity 8 (i.e. high intensity). Again, we observed that both mutant and sibling larvae responded to the tap with a burst of movement, and that both mutant lines elicited motor responses equal to or greater than that of their siblings at 5dpf (Fig. 5B,D). Unexpectedly, we found that the *vps11(plt)* larvae moved a greater distance than their siblings in response to the tap stimulus (Fig. 5B).

**Figure 6:**
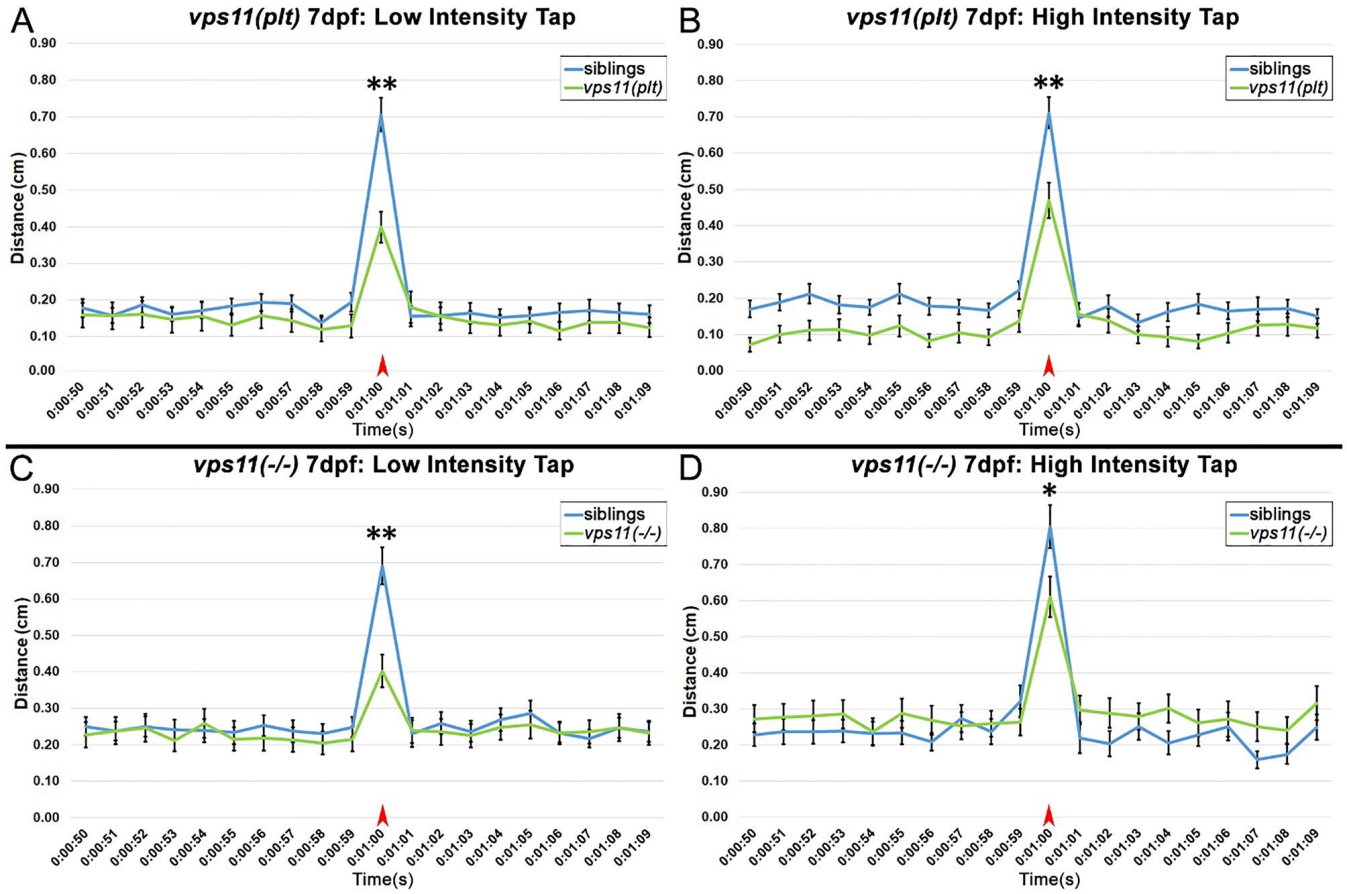
*vps11(plt)* and *vps11*(−/−) mutants at 7dpf show significantly reduced distance travelled in response to both low and high intensity acoustic/tap stimuli. (**A**–**D**) Line graphs representing distance travelled by larvae at 7dpf before and after an acoustic/tap stimulus (red arrow). (**A**) Response of *vps11(plt)* (green; n = 69) and sibling larvae (blue; n = 77) to a low intensity stimulus. (**B**) Response of *vps11(plt)* (green; n = 71) and sibling larvae (blue; n = 73) to a high intensity stimulus. (**C**) Response of *vps11*(−/−) (green; n = 54) and sibling larvae (blue; n = 54) to a low intensity stimulus. (**D**) Response of *vps11*(−/−) (green; n = 48) and sibling larvae (blue; n = 47) to a high intensity stimulus. *P < 0.05 **P < 0.01. Error bars indicate SEM.

Next, we repeated the acoustic/tap test with a new cohort of larvae at 7dpf. Starting with a single-tap stimulus at the low intensity, we observed that both *vps11(plt)* and *vps11*(−/−) larvae travelled a significantly lower distance compared with their respective siblings in response to the tap (Fig. 6A,C). Next, we tested the response to the highest intensity tap, and again found that both mutant larvae travelled a significantly lower distance compared with their respective siblings in response to the tap (Fig. 6B,D). Collectively, these data suggest that both *vps11(plt)* and *vps11*(−/−) mutants exhibit progressive sensorimotor deficits from 5 to 7dpf in response to a startle stimulus that was independent of the visual system.

### ***vps11(plt)*** and ***vps11***(−/−) mutants show habituation responses to multiple acoustic stimuli

**Figure 7:**
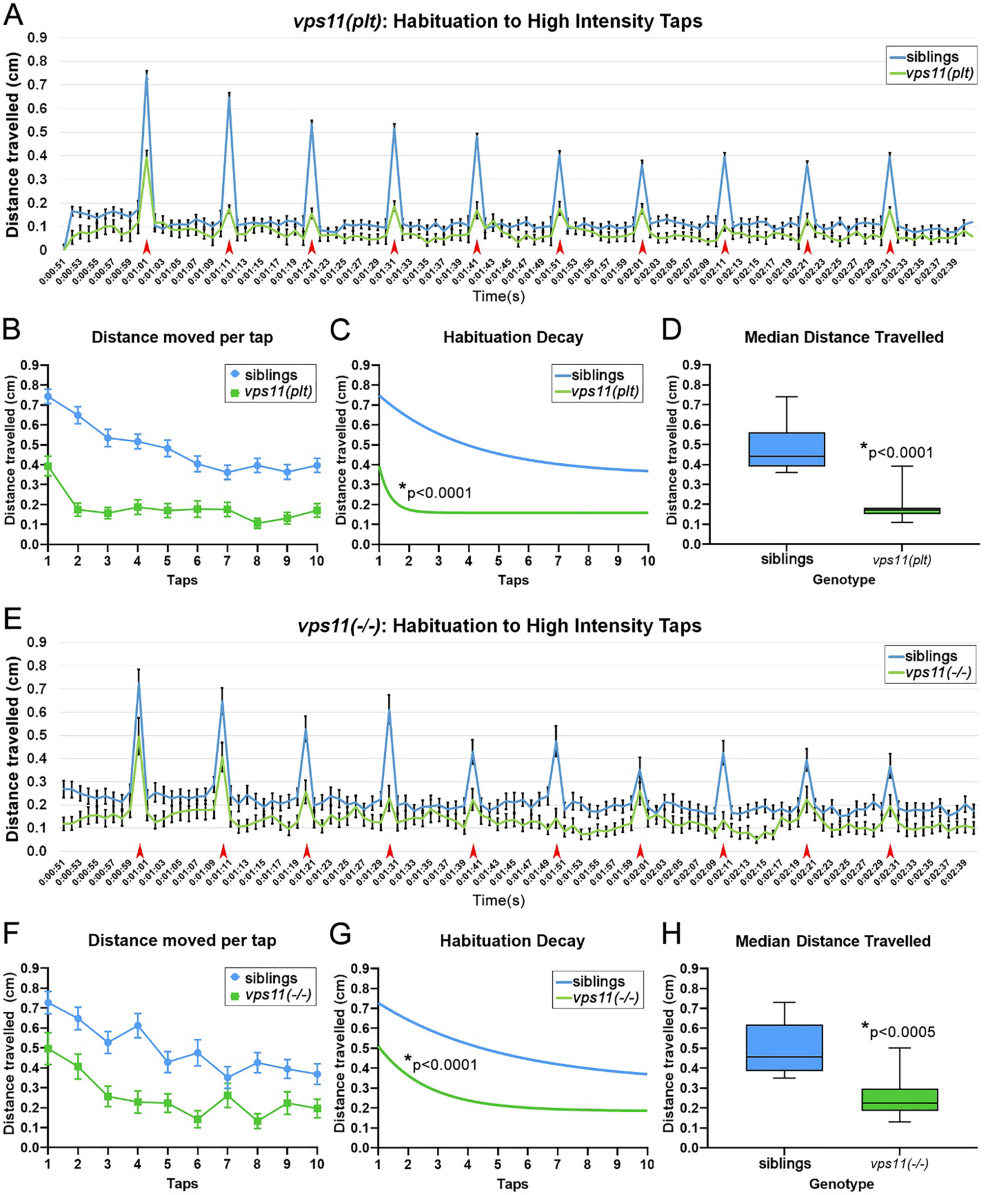
*vps11(plt)* and *vps11*(−/−) mutants at 7dpf show a significantly reduced habituation response to multiple high intensity acoustic/tap stimuli compared with their siblings. (**A**) Line graph representing distance travelled by larvae at 7dpf during the complete high intensity multiple tap paradigm comprising 10 high intensity taps (red arrows) with 10 s inter-stimulus intervals (sibling larvae in blue, *vps11(plt)* in green). (**B**) Graph representing average distance moved by sibling (blue; n = 68) and *vps11(plt)* (green; n = 64) larvae at each individual tap stimuli. (**C**) First order exponential decay curve representing average distance travelled by sibling (blue; n = 68) and *vps11(plt)* (green; n = 64) larvae at each individual tap stimuli *P < 0.0001. (**D**) Box-plots representing the average distance travelled by sibling (blue; n = 68) and *vps11(plt)* (green; n = 64) larvae at all 10 tap stimuli. *P < 0.0001. (**E**) Line graph representing distance travelled by larvae at 7dpf during the complete high intensity multiple tap paradigm comprising 10 high intensity taps (red arrows) with 10 s inter-stimulus intervals (sibling larvae in blue, *vps11*(−/−) in green). (**F**) Graph representing average distance moved by sibling (blue; n = 48) and *vps11*(−/−) (green; n = 44) larvae at each individual tap stimuli. (**G**) First order exponential decay curve representing average distance travelled by sibling (blue; n = 48) and *vps11*(−/−) (green; n = 44) larvae at each individual tap stimuli *P < 0.0001. H. Box-plots representing the average distance travelled by sibling (blue; n = 48) and *vps11*(−/−) (green; n = 44) larvae at all 10 tap stimuli *P < 0.0005. Error bars indicate SEM.

**Figure 8:**
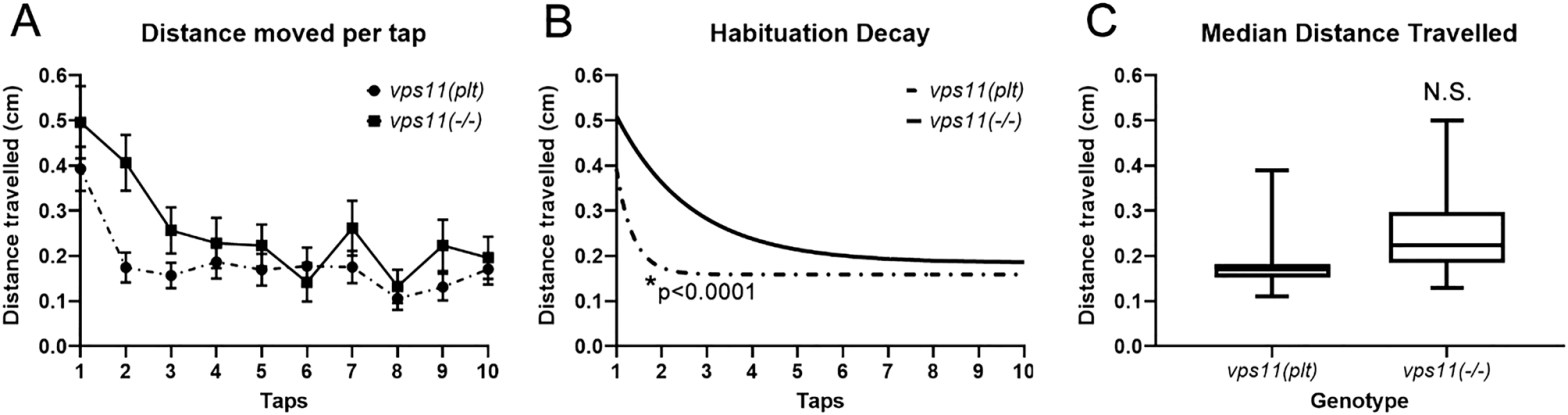
*vps11(plt)* and *vps11*(−/−) mutants show different rates of habituation response to multiple high intensity acoustic/tap stimuli. (**A**) Graph representing average distance moved by *vps11(plt)* (dashed line; n = 64) and *vps11*(−/−) (solid line; n = 44) larvae at each individual tap stimuli. B. First order exponential decay curve representing average distance travelled by *vps11(plt)* (dashed line; n = 64) and *vps11*(−/−) (solid line; n = 44) larvae at each individual tap stimuli *P < 0.0001. (**C**) Box-plots representing the average distance travelled by *vps11(plt)* (n = 64) and *vps11*(−/−) (n = 44) larvae at all 10 tap stimuli. Error bars indicate SEM.

Finally, as an initial assessment of higher circuit development (i.e. short-term memory), we tested the habituation response in the mutant lines in response to series of acoustic taps. Similar to a recent report^14^, we found that 10 consecutive high intensity taps at 10-s intervals resulted in a robust habituation response in sibling larvae at 7dpf (Fig. 7A). Next, we tested both *vps11(plt)* (Fig. 7A–D) and *vps11*(−/−) mutants (Fig. 7E–H) at 7dpf using this habituation paradigm and found that both mutant lines responded to each tap with a burst of movement followed by a return to baseline and that both mutant lines exhibited a habituation response to these series of taps (Fig. 7A–B,E–F). In addition, we found that both mutant lines habituated at a significantly faster rate than their siblings, and had a significantly lower median distance travelled (Fig. 7C–D, 7G-H). Interestingly, when we compared the *vps11(plt)* and *vps11*(−/−) mutant datasets to each other, we found no difference in the median distanced travelled, but a significant difference in the habituation decay curves between the mutants (Fig. 8). Specifically, we observed that the *vps11(plt)* mutants habituated significantly faster than the *vps11*(−/−) mutants (Fig. 8B). Together, we observed that while both mutant lines exhibited habituation responses to a series of acoustic stimuli, the dynamics of the response were not equivalent.

## Discussion

The mutations in human *VPS11(C846G)*, zebrafish *vps11(plt),* and *vps11*(−/−) result in increasingly-truncated protein products (Fig. 1). The missense mutation *VPS11:C846G* associated with gLE in human patients was predicted to disrupt its C-terminal RING-H2 domain (Fig. 1). This domain functions in estrogen receptor-α, progesterone receptor, Wnt, NFκB and FoxO signaling pathways using its E3 ubiquitin ligase function^17^. Interestingly, protein structure analysis of the RING-H2 domain indicated that the truncated VPS11:C846G structure is not misfolded in vitro^6^. In addition, the location of the truncation predicted that VPS11:C846G could interact with VPS18 and other associated binding partners (Fig. 1;^18,19^. Consistent with this, Zhang et al., 2016^6^ reported that mutant VPS11(C846G) protein bound wild-type VPS18 in vitro, but at a reduced level, raising the possibility of hypomorphic function for the VPS11(C846G) protein. The zebrafish *vps11(plt)* mutation results in an even more severe truncation, which is predicted to disrupt Vps11(plt) binding to Vps18 and Vps39/Vps3, but not Vps8 (Fig. 1). However, despite our ability to detect *vps11(plt)* transcript, it is unclear whether the mutant protein is translated, stable, and/or provides any function. Furthermore, if the Vps11(plt) peptide is made, it could act in a dominant-negative function by sequestering wild-type Vps8, but not participating in the larger HOPS/CORVET complexes. We think this is unlikely, as we never observe any phenotypes in the heterozygotes, including OKR (Suppl. Fig. 3), but given these possibilities, we sought to compare the visuomotor function of *vps11(plt)* mutant fish to a *vps11*(−/−) null mutant. We first created a *vps11*(−/−) mutant zebrafish using TALENs with an indel in exon 1 of *vps11* (Fig. 1). Phenotypic analysis indicated that these mutants displayed the classic pathologies of hypopigmentation, hypomyelination, and CNS apoptosis (Suppl. Fig. 1). Next, we used these animals to ask whether these mutants had similar or more/less severe visuomotor deficits than the *vps11(plt)* mutants.

Zebrafish larvae display a range of distinct behavioral swimming patterns in response to environmental factors, including the presence or absence of light^16,20,21^. Given a choice between light and dark environments, adult zebrafish show a preference for a dark environment^22^, whereas larvae show a strong avoidance of a dark environment^23^. However, when zebrafish larvae are placed in a light environment with decreasing light over time, larvae show an increase in swimming turn angles^24^. As dark conditions persist, this hyperactivity of turn angles plateaus, and larval fish begin to swim normally^16,24^. Once acclimated to dark conditions, larval fish exhibit a “freeze” response to the sudden introduction of light, decreasing their swimming distance and velocity^16^. Based on these previously established paradigms, we designed an alternating light/dark behavioral assay that included a 12-min period of dark acclimation followed by alternating 3-min intervals of light and dark (Suppl. Fig. 2). Using this approach, we showed that wild-type (AB) larva at 7dpf showed a robust “peak and valley” movement response to dark and light conditions, respectively (Suppl. Fig. 2). Importantly, using the Noldus DanioVision behavioral tracking system, we showed that this response was highly reproducible, giving us a robust and unbiased assay to test our mutant zebrafish lines.

Next, we observed that *vps11(plt)* and *vps11*(−/−) larva showed a progressive loss of normal visuomotor response to alternating light/dark conditions (Figs. 2, 3). At 5dpf, both mutant lines showed no significant differences in distance moved or velocity in the dark condition compared with their corresponding siblings (Figs. 2C–D & 3C–D). In the light condition, the 5dpf *vps11(plt)* mutants showed a significant increase in distance moved and velocity compared with their siblings. While this finding is somewhat counterintuitive for a hypomyelination pathology, this result may represent a partial failure or delay in the “freeze” response to light onset. Even with this finding, both mutant lines showed normal movement patterns compared with their siblings at 5dpf. In contrast, at 7dpf, both *vps11(plt)* and *vps11*(−/−) larva showed significantly reduced movement compared with their siblings (Fig. 2F,F). In the *vps11*(−/−) larva, movement in both the light and dark conditions was significantly lower (Fig. 3G–H), whereas in *vps11(plt)* mutants, only movement in the dark was significantly lower (Fig. 2G–H). The difference in movement between the mutants and siblings at 7dpf was largely due to an increase in distance moved and velocity on the part of sibling animals, and the corresponding failure of the mutant animals to develop a similar increase in movement. The increase in sibling movement at 7dpf correlates with an increase in myelination during zebrafish CNS development and an established increase in swimming patterns^20,21,25^. Movement of the mutant larva increased from 5 to 7dpf, but not at to the level observed in sibling animals. This result is consistent with the hypomyelination phenotype observed in these mutants (Suppl. Fig. 1) and our previous finding that *vps11(plt)* larva show reduced myelin basic protein (MBP) expression surrounding the Mauthner axons in the ventral hindbrain at 7dpf^6^.

Collectively, we show that *vps11(plt)* and *vps11*(−/−) larva respond to light and dark, but show a progressive loss of visuomotor responses from 5 to 7dpf. However, detection of light and dark does not equate to visual acuity. In humans, intrinsically photosensitive ganglion cells (pRGCs) function in the light-induced pupillary light reflex, and some studies have suggested that they may also play a role in circadian rhythms and as a rudimentary light detector^26^. In zebrafish, which are semi-transparent during development, neurons in the hindbrain show non-visual sensation^27^, and zebrafish larvae lacking eyes and pineal gland still respond to light and perform light seeking behavior^28^. Therefore, while the *vps11(plt)* and *vps11*(−/−) larva clearly responded to light and dark, these experiments did not give us any insight into their visual acuity.

The optokinetic response (OKR) has become an invaluable tool for assessing visual acuity in children with neurological disorders^29^. Alert neonates can track large, slow-moving objects within the first few days of life^30^ and nystagmus can be diagnosed within the first few weeks of life by OKR^31–33^. In zebrafish, the visual system is sufficiently developed by 5dpf to measure the OKR, and this approach has been used to test the visual acuity of a large number of retinal degeneration mutants^34–38^. We first used traditional OKR to assess visual acuity of both *vps11(plt)* and *vps11*(−/−) mutants at 5dpf. Under conditions of constant velocity and constant spatial frequency of the black and white grating, the 5dpf mutant larvae showed a severely impaired response at 100% contrast (Fig. 4A). Next, we repeated the OKR under modified conditions based on the possibility that the hypomyelination pathology of the mutant fish would limit their ability to track the moving stimulus at the default rotating velocity of 7.5 degree/sec. Under conditions of a constant contrast and spatial frequency, we instead changed the stimulus velocity of the moving drum. However, we again found that the response of *vps11(plt)* and *vps11*(−/−) mutants to this modified stimulus was near baseline at all velocities tested. (Fig. 4B). These experiments suggest that both mutants are significantly visually impaired by 5dpf (i.e. not able to visually track and process an image).

A previous report showed a retinal degeneration phenotype in *vps11(plt)* mutants at 5dpf^10^, which could account for the OKR defect we observed in *vps11(plt)* and *vps11*(−/−) mutants. Due to the small number of reported cases of children diagnosed with the *VPS11(C846G)* mutation, it is currently unclear whether affected individuals also develop retinal degeneration; however, Zhang et al., 2016^6^ reported that the loss of vision in their cohort of 5 patients was likely due to cortical blindness/optic atrophy, and not retinal degeneration. This issue will likely be clarified as more clinical case reports emerge. The early retinal degeneration phenotype in the *vps11(plt)* mutants may result from the constraints of the rapid embryonic development of zebrafish, driving an accelerated and/or exacerbated neuronal degeneration pathology. Future work on *Vps11* mutant mice, which develop slower than zebrafish during early development, may give some insight into any underlining contribution of retinal degeneration to Vps11-dependent vision loss.

Paired with MRI, the stagnation, delay, or regression in developing motor skills are critical for definitive diagnosis of gLE^2^. Consistent with this, Zhang et al., 2016^6^ reported that all patients with the *VPS11(C846G)* mutation initially presented with low tone and/or delay in motor skill development. As a first attempt to test the sensorimotor function of *vps11(plt)* and *vps11*(−/−) mutants separate from the visual system, we utilized an acoustic/tap startle response at low and high intensities. The Noldus DanioVision System comes with 8 preset intensities for the tap response, which is initiated as a physical tap on the bottom of the stage. We first tested the minimum intensity that elicited a robust and significant response over baseline movement in 7dpf AB wild-type larvae. We hypothesized that this “low intensity tap” might be below the sensory threshold to elicit a response in the mutant animals. We first tested the mutants at 5dpf. Unexpectedly, both the *vps11(plt)* and *vps11*(−/−) mutants responded to the low intensity tap with a burst of movement that was not significantly lower than their siblings at 5dpf (Fig. 5A,C). When we repeated the test using the highest intensity tap, we found that *vps11*(−/−) mutants moved a similar distance to their siblings (Fig. 5D), and that *vps11(plt)* mutant surprisingly moved an even greater distance than their siblings (Fig. 5B). Next, we repeated the single-tap paradigm on 7dpf mutants and found that both mutant lines responded to the low and high intensity tap, but moved a significantly shorter distance than their siblings (Fig. 6). Interestingly, while both mutants had an impaired response at 7dpf, they were still able to distinguish between low and high intensity taps. Together, these findings demonstrate a progressive loss of sensorimotor function in *vps11(plt)* and *vps11*(−/−) mutant zebrafish that is consistent with the progressive impairment of motor function in patients with gLE^2^.

Caution should be given to equating the acoustic/tap stimulus in zebrafish to acoustic, startle-responses used to test hearing loss in human infants. In zebrafish, the acoustic/tap must reverberate through the water and would therefore be perceived by their lateral line system. This network of sensory hair neurons on the body flank of fish is used to sense movement in the surrounding water^39^. It is well-established that motor responses to acoustic stimuli in larval and adult zebrafish are mediated through the combination of the lateral line hair cells and the highly-myelinated Mauthner neurons in the hindbrain^40–42^. Chemical ablation of the hair cells or loss of the Mauthner neurons results in a latency in escape response to acoustic stimuli^42,43^. We previously showed that 7dpf *vps11(plt)* larvae display significant hypomyelination of Mauthner neurons, which may account for their reduced startle response to the acoustic/tap stimulus^6^. Finally, it should be noted that even in human patients with gLE, including patients with the *VPS11(C846G)* mutation, hearing loss is highly variable in severity^6^.

A recent study demonstrated that wild-type zebrafish larvae exhibit a classic habituation response to multiple acoustic/tap stimuli by 5dpf^14^, reinforcing previous work from the 1970s that first used zebrafish to study this basic form of learning^44,45^. As an initial assessment of higher circuit development in our mutant lines, we tested their habituation response to series of acoustic taps at 7dpf (Fig. 7). A number of interesting findings emerged from this set of experiments. First, we observed that both *vps11(plt)* and *vps11*(−/−) mutants responded to each tap with a burst of movement followed by a return to baseline (Figs. 7A,E). Hyperactivity, sporadic movements, or failure to respond was not observed. However, we did observe that both mutant lines habituated at a significantly faster rate than their siblings (Fig. 7C,G). Finally, when we compared the *vps11(plt)* and *vps11*(−/−) mutant datasets to each other, we found that the *vps11(plt)* mutants habituated significantly faster than the *vps11*(−/−) mutants (Fig. 8B). It is unclear what may account for this difference, but it should be noted that we observed several unexpected findings from the *vps11(plt)* mutants in our previous behavioral paradigms. As noted above, under two conditions, the *vps11(plt)* mutants actually moved more than their siblings in response to light stimulation and a single acoustic tap (Figs. 2C–D, 5B). We cannot account for the difference between the mutants based on obvious variables; both lines have been on the same AB background strain for over five years, multiple different carrier pairs were used to generate larva for these studies, and the overall sample size is high. We can therefore only discuss potential reasons that could be used to drive future explorations. It is possible that differences in protein structure and HOPS/CORVET complex formation accounts for these differences. If *vps11(plt)* mutants make a truncated Vps11(plt) protein, then it is possible that cellular energy used to constantly degrade *vps11(plt)* mutant mRNA and/or protein could result in greater “stress” than simply not have a protein in the *vps11*(−/−) null animals. It is also possible that the *vps11(plt)* mutants do not habituate faster in the classical sense, but instead have a higher level of motor fatigue. Regardless of the mechanism, the outcome on the *vps11(plt)* animals is subtle, as we have not noted any other pathological differences between the *vps11(plt)* and *vps11*(−/−) mutants to present. Future work will be needed to determine whether the differences we reported here persist with higher-level cognitive behavioral tests (i.e. long-term memory).

Collectively, we show for the first time a progressive loss of visuomotor responses in a vertebrate Vps11 mutant model. These data are consistent with the gradual loss of visual acuity and motor tone in human gLE patients with the *VPS11:C846G* mutation. We suggest that the mutants and behavioral assays described here could be a valuable model system in which to test potential pharmacological interventions for gLE.

## Supplementary Information

Supplementary Information.
