# Supplementary material for: Vision and sensorimotor defects associated with loss of Vps11 function in a zebrafish model of genetic leukoencephalopathy
Source: Sci Rep. 2022 Mar 3;12:3511. doi: 10.1038/s41598-022-07448-1 (PMC8894412; doi:10.1038/s41598-022-07448-1)
Supplement: Supplementary file 1 — Supplementary Information. [file 41598_2022_7448_MOESM1_ESM.docx]

**Vision and sensorimotor defects associated with loss of Vps11 function in a zebrafish model of genetic leukoencephalopathy**

**Shreya Banerjee^1^, Lillian E. Ranspach^1^, Xixia Luo^1^, Lauren T. Cianciolo^2^, Joseph Fogerty^2^, Brian D. Perkins^2^, Ryan Thummel^1*^**

**SUPPLEMENTARY FIGURES**

Figure S1:


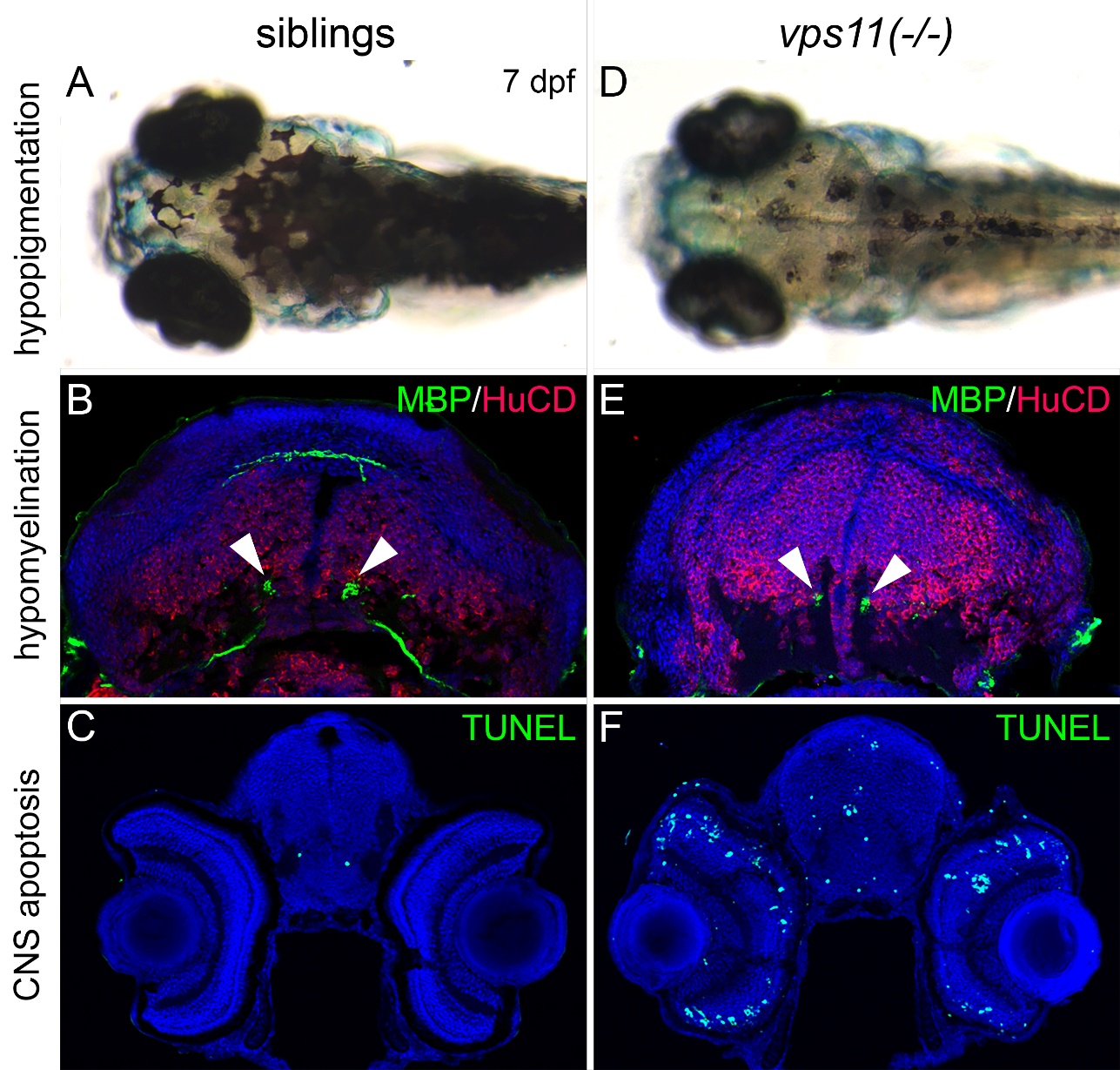


**Figure S1: New *vps11(-/-)* larva show classic pathologies similar to *vps11(plt)* mutants.**

A-C. Wild-type siblings of *vps11(-/-)* larva at 7 dpf show normal pigmentation (A), myelination of the Mauthner axons in the hindbrain as determined by anti-Myelin Basic Protein immunolocalization (B, arrowheads), and minimal apoptosis in the brain and retina as determined by TUNEL analysis (C, green). D-F. *vps11(-/-)* mutant larva at 7 dpf show hypopigmentation of body melanophores (D), reduced myelination (E, arrowheads), and large numbers of apoptotic cells in the brain and retina (F).

Figure S2:


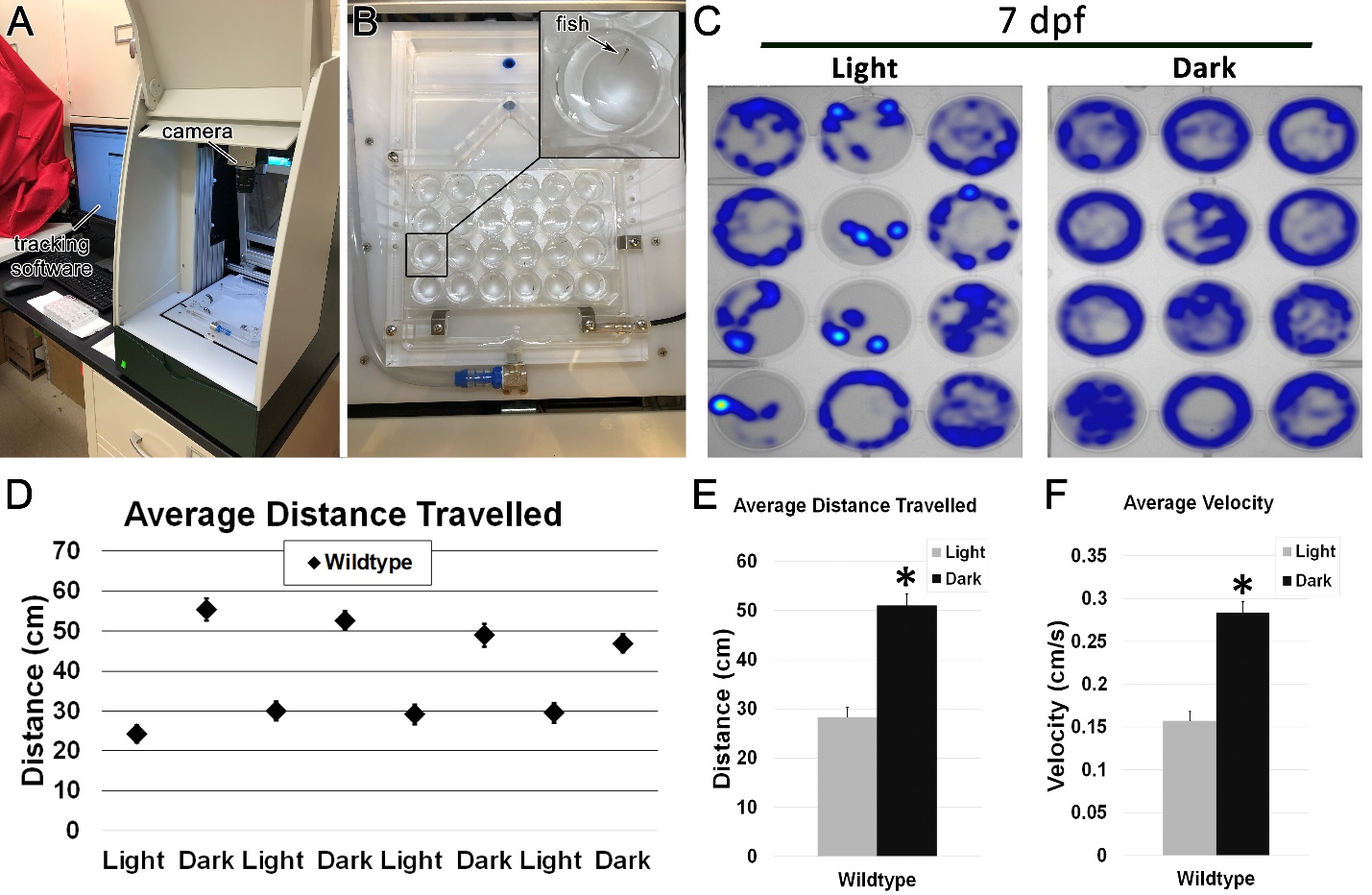


**Figure S2: Noldus DanioVision and EthoVision XT tracking software can be used to track behavioral responses of zebrafish larvae during alternating cycles of light and dark periods.**

A. Noldus DanioVision Observation Chamber set-up containing Basler Gen1 tracking camera linked to EthoVision XT13 tracking software. B. Individual fish wild-type (AB) larva at 7 days post fertilization (dpf) are placed in a single well of a 24-well plate. The plate is placed within the Observation Chamber and subjected to a steady stream of flowing water to maintain constant temperature during experimentation. C. Heat maps generated by the EthoVision XT13 tracking software displaying total distance moved by individual larvae (represented in blue) during 3-minute periods of light and dark. D. Graph representing average distance travelled in 4 alternating light-dark cycles of 3 minutes each. E. Bar graph representing average distance travelled in light (grey) and dark (black) periods. F. Bar graph representing average velocity of wild-type larvae in light (grey) and dark (black) periods. n = 48 wild-type larvae at 7 dpf. * p < 0.01. Error bars indicate SEM.

Figure S3:


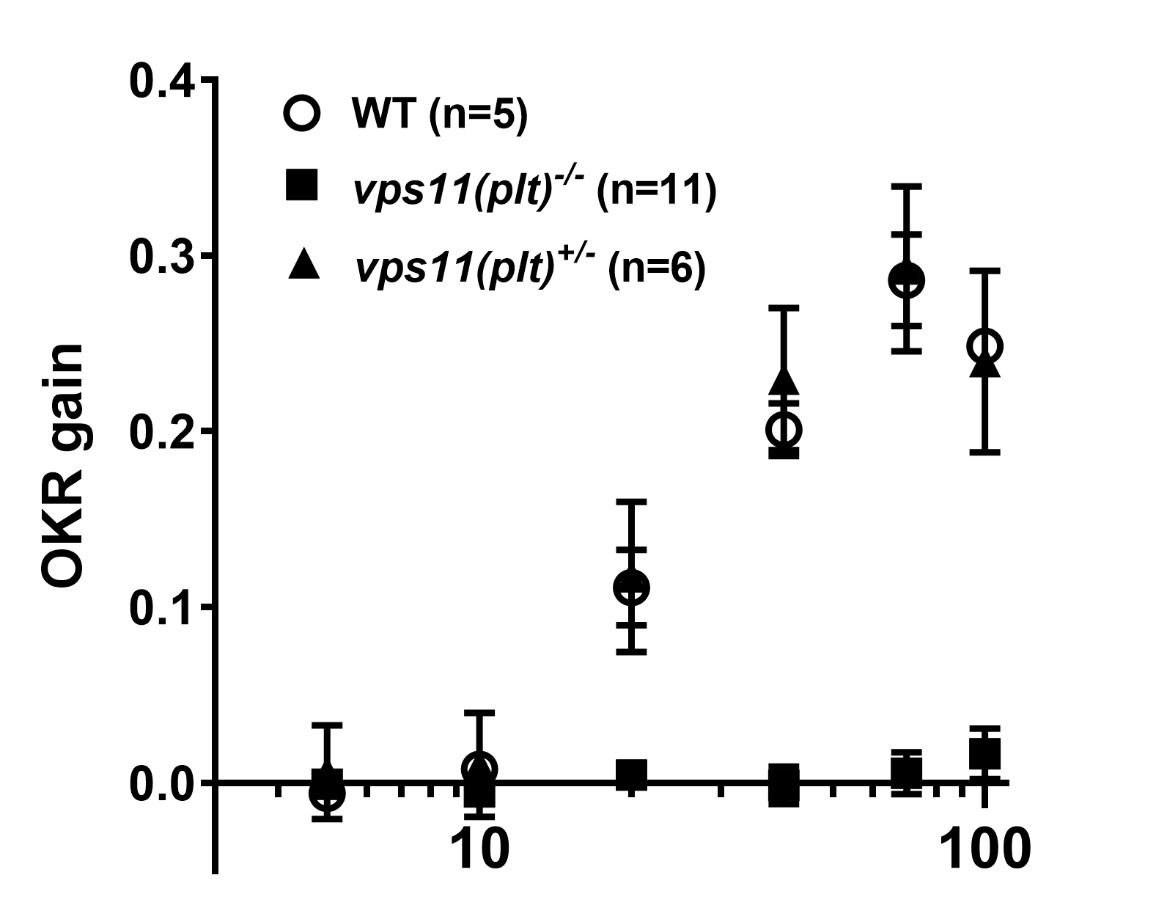


**Figure S3: No differences in visual function between *vps11(plt)* heterozygotes and their wild-type siblings.**

Optokinetic response of *vps11(plt)* homozygotes (-/-), heterozygotes (+/-), and wild-type siblings (WT) at 5dpf. No significant difference was found in OKR gain (y axis) between the heterozygotes and wild-type siblings at any percentage of contrast sensitivity (x axis).

**SUPPLEMENTARY MATERIALS**

**Immunohistochemistry**

Immunohistochemistry was performed as described ^1^ on frozen sections from *vps11(-/-)* mutant and siblings collected at 7dpf. Primary antibodies included: rabbit anti-mbp (1:200; gift from Dr. Bruce Appel, University of Colorado), mouse anti-HuC/HuD monoclonal anti-body (1:50, clone 16A11, product# A-21271, Thermo Fisher Scientific, USA). AlexaFluor goat anti-rabbit IgG 488 (1:500) and goat anti-mouse IgG 594 (1:500) was used as a secondary antibody (Invitrogen-Molecular Probes, Eugene, OR). Nuclei were labeled (TO-PRO-3; Invitrogen-Molecular Probes) at a 1:750 dilution in 1 PBS/0.05% Tween-20. Tissue sections were mounted with Prolong Gold Antifade Reagent (Cell Signaling Technology). Single plane confocal images were acquired using identical confocal settings on a Leica TCS SP8 confocal microscope.

**Terminal Deoxynucleotidyl Transferase dUTP Nick End labeling (TUNEL) analysis**
TUNEL was performed using tissue sections processed for standard immunohistochemistry
Briefly, tissue sections were washed in 1xPBS for 20min, permeabilized with ice-cold buffer of 0.1% NaCitrate/0.1% Triton X-100/1XPBS for 2min, and washed in 1xPBS for 5min at RT. Next, sections were incubated in 100μL labeling buffer (ApoAlert DNA fragmentation kit; Clontech International) for 10min at RT, followed by humidified incubation with 50μL of labeling mix (48μL labeling buffer, 1μL of 1mM biotinylated dNTPs (New England Biolabs) and 1μL of TdT enzyme (45U/μL; ApoAlert DNA fragmentation kit; Clontech International), at 37°C, 1–2hr. The reaction was stopped with a 15min RT wash with 150μL of 2XSSC. Tissue sections were washed in 1XPBS and then incubated with StrepTavidin conjugated to AlexaFluor 488 (1:200, Invitrogen-Molecular Probes, Eugene, OR) and TO-PRO-3 (1:750, Invitrogen-Molecular Probes) diluted with 1XPBS for 1hr in the dark, washed with PBS, and mounted with Prolong Gold Antifade Reagent (Cell Signaling Technology). Single plane confocal images were acquired using identical confocal settings on a Leica TCS SP8 confocal microscope.

1 Thomas, J. L. *et al.* The loss of vacuolar protein sorting 11 (vps11) causes retinal pathogenesis in a vertebrate model of syndromic albinism. *Invest Ophthalmol Vis Sci* **52**, 3119-3128, doi:10.1167/iovs.10-5957 (2011).
